# Agmatine augmentation in treatment-resistant obsessive-compulsive disorder: a prospective open-label case series

**DOI:** 10.3389/fpsyt.2026.1745041

**Published:** 2026-02-04

**Authors:** Joshua D. Salvi

**Affiliations:** 1 Center for OCD and Related Disorders, Department of Psychiatry, Massachusetts General Hospital, Boston, MA, United States; 2 Department of Psychiatry, Harvard Medical School, Boston, MA, United States

**Keywords:** agmatine (PubChem CID: 199), agmatine sulfate, anxiety, OCD (obsessive-compulsive disorder), psychopharmacology

## Abstract

**Importance:**

Obsessive-compulsive disorder (OCD) remains treatment-resistant in 40-60% of patients despite adequate trials of serotonin reuptake inhibitors and cognitive behavioral therapy. Glutamatergic dysfunction in cortico-striato-thalamo-cortical circuits has emerged as a key pathophysiologic mechanism, prompting investigation of glutamatergic modulators as augmentation strategies.

**Objective:**

To evaluate the safety, tolerability, and preliminary efficacy of agmatine sulfate augmentation in treatment-resistant OCD.

**Design:**

Prospective open-label case series conducted between May 2025 and November 2025.

**Setting:**

Outpatient psychiatry practice.

**Participants:**

Five adults (ages 28–54 years) with treatment-resistant OCD who had failed multiple serotonin reuptake inhibitor trials and at least one augmentation strategy.

**Intervention:**

All patients were treated with agmatine sulfate initiated at 650 mg daily, increased to 1300 mg daily after one week, as augmentation to stable doses of selective serotonin reuptake inhibitors.

**Main outcomes and measures:**

We measured the OCD severity using the Yale-Brown Obsessive Compulsive Scale (Y-BOCS) scores assessed at baseline and multiple time points over 112 days. Primary outcome was change in Y-BOCS score from baseline.

**Results:**

Exponential decay modeling revealed gradual symptom reduction with half-life of 16.4 days. Two of five patients (40%) demonstrated clinically meaningful improvement (≥25% Y-BOCS reduction). Agmatine was well-tolerated with no discontinuations due to adverse effects. Mild transient gastrointestinal symptoms occurred in two patients.

**Conclusions and relevance:**

This case series provides preliminary, early evidence supporting agmatine’s safety and potential efficacy as augmentation therapy in treatment-resistant OCD. The gradual response pattern and 40% responder rate warrant controlled trials to definitively establish efficacy and identify predictors of treatment response.

## Introduction

Obsessive-compulsive disorder (OCD) affects 2-3% of the population and ranks among the leading causes of disability worldwide (1). Despite decades of research establishing selective serotonin reuptake inhibitors (SSRIs) and cognitive behavioral therapy with exposure and response prevention as first-line treatments, 40-60% of patients fail to achieve adequate symptom control (2, 3). Among treatment-resistant patients, established augmentation strategies—primarily second-generation antipsychotics—provide meaningful benefit to only 28-33% of cases (4), leaving a substantial population with limited therapeutic options and persistent functional impairment.

Converging evidence from neuroimaging, cerebrospinal fluid analyses, and genetic studies implicates glutamatergic dysfunction in OCD pathophysiology (5, 6). Magnetic resonance spectroscopy demonstrates elevated glutamate concentrations in the caudate nucleus and anterior cingulate cortex of OCD patients, while genetic associations have been identified for genes encoding key glutamate system components including SLC1A1 and GRIN2B (7). These findings have prompted investigation of glutamatergic modulators as novel augmentation strategies.

A recent comprehensive meta-analysis by Coelho and colleagues demonstrated large effect sizes (Cohen’s d = -0.80) for glutamatergic medications across obsessive-compulsive and related disorders, with OCD-specific analyses revealing mean Y-BOCS reductions of 4.17 points (8). However, substantial heterogeneity (I² = 88%) and variable response rates of 25-50% highlight ongoing challenges in glutamatergic augmentation strategies. Individual agents including memantine, riluzole, and N-acetylcysteine show mixed results across trials (9, 10), underscoring the need for investigation of additional compounds within this mechanistic class.

Agmatine, an endogenous polyamine synthesized from L-arginine by mitochondrial arginine decarboxylase, represents a novel therapeutic candidate based on its unique pharmacological profile (11). The compound functions as a selective antagonist of GluN2B-containing NMDA receptors while simultaneously activating AMPA receptors and mTOR signaling pathways (12, 13). Preclinical studies demonstrate anti-compulsive effects in marble-burying behavior models, with agmatine significantly reducing compulsive-like behaviors without affecting locomotor activity (14). Pharmacokinetic studies confirm adequate central nervous system penetration following oral administration, with sustained brain tissue residence despite short plasma half-life (15).

Human safety data, though limited, support agmatine’s tolerability. A study in patients with lumbar radiculopathy documented minimal adverse effects at doses up to 3.56 g daily for 21 days, with only mild gastrointestinal symptoms in three patients at the highest dose. Despite strong mechanistic rationale and established safety, no clinical studies have examined agmatine in OCD populations.

This case series reports preliminary safety, tolerability, and efficacy data for agmatine sulfate augmentation in five patients with treatment-resistant OCD. All patients received agmatine 1300 mg daily as augmentation to stable SSRI therapy, with Y-BOCS scores assessed prospectively over 150 days. We elected for a lower dose of agmatine at this juncture to determine whether such a low dose could be adequate for improving outcomes in treatment-resistant OCD. We aimed to establish preliminary evidence regarding agmatine’s therapeutic potential, characterize the time course of clinical response, and identify safety concerns that might preclude larger controlled trials.

## Methods

### Patient selection and design

This prospective open-label case series was conducted in an outpatient psychiatry practice between May 2025 and November 2025. Five adults with treatment-resistant OCD were enrolled. Inclusion criteria included: (1) primary diagnosis of OCD per DSM-5 criteria; (2) baseline Y-BOCS score ≥16; (3) inadequate response to at least two adequate SSRI trials (≥12 weeks at maximum tolerated dose); (4) failure of at least one augmentation strategy; and (5) stable medication regimen for ≥8 weeks prior to agmatine initiation. Exclusion criteria included active substance use disorders, psychotic disorders, and pregnancy or breastfeeding. Subjects were required to maintain the same dose of concurrent medications throughout the duration of the study.

### Intervention

Agmatine sulfate was initiated at 650 mg daily (650 mg once daily), increased to 1300 mg daily (650 mg twice daily) after one week (Day 0). Agmatine sulfate was manufactured by Swanson Health Products, produced through a GMP-certified facility, which requires a Certificate of Analysis for purity testing, potency, pathogenic bacteria, fungal counts, and heavy metals (lead, mercury, cadmium, and arsenic). Patients maintained stable doses of concurrent psychotropic medications throughout the study period. Agmatine sulfate was obtained from pharmaceutical-grade supplement sources.

### Outcome measures

The Yale-Brown Obsessive Compulsive Scale (Y-BOCS), a clinician-administered 10-item scale assessing obsession and compulsion severity (range 0-40, higher scores indicating greater severity), served as the primary outcome measure. Y-BOCS assessments were conducted at baseline, day 14, day 28, day 56, day 84, day 112, and day 150. Clinical response was defined as ≥25% reduction in Y-BOCS score from baseline. Adverse effects were monitored via structured interview at each assessment.

### Statistical analysis

Y-BOCS scores were analyzed using exponential decay modeling: Y = (Y_0_ - b) × exp(-µ × X) + b, where Y0 represents baseline, b represents asymptotic minimum, and µ represents decay constant. Though descriptive given the small sample size, half-life was calculated as ln(2)/µ. Normality was assessed using the D’Agostino-Pearson omnibus test. Data are presented as means with standard error of the mean (SEM).

## Results

### Patient characteristics

Five patients (3 male, 2 female; mean age 39.6 years, range 28-54) with treatment-resistant OCD participated (**Table 1**). All patients had failed multiple SSRI trials, with four having failed clomipramine or second-generation antipsychotic augmentation. Predominant symptom dimensions included contamination (n=3), symmetry/exactness (n=4), and checking (n=1). All patients had psychiatric comorbidities, most commonly major depressive disorder (n=5).

**Table 1:** Patient characteristics and treatment history.

Patient	Age/Sex	OCD symptom dimensions	Comorbidities	Concurrent medications	Prior failed treatments
A	34/M	Contamination	Trichotillomania, MDD	Sertraline 200 mg/d	Multiple SSRIs, clomipramine, SGA augmentation, NAC 2400 mg/d
B	28/F	Checking, relationship, symmetry	GAD, MDD	Escitalopram 20 mg/d	Multiple SSRIs, SGA augmentation (not tolerated)
C	54/M	Symmetry, exactness, reassurance-seeking	Persistent depressive disorder, insomnia	Sertraline 250 mg/d, aripiprazole 2 mg/d	Multiple SSRIs
D	43/M	Contamination	BDD, MDD	Escitalopram 30 mg/d, buspirone 10 mg BID	Multiple SSRIs, clomipramine
E	39/F	Symmetry, exactness, contamination	Emetophobia, MDD	Fluoxetine 80 mg/d	Multiple SSRIs, aripiprazole, memantine

BDD, body dysmorphic disorder; GAD, generalized anxiety disorder; MDD, major depressive disorder; NAC, N-acetylcysteine; SGA, second-generation antipsychotic; SSRI, selective serotonin reuptake inhibitor.

### Clinical outcomes

**Figure 1:**
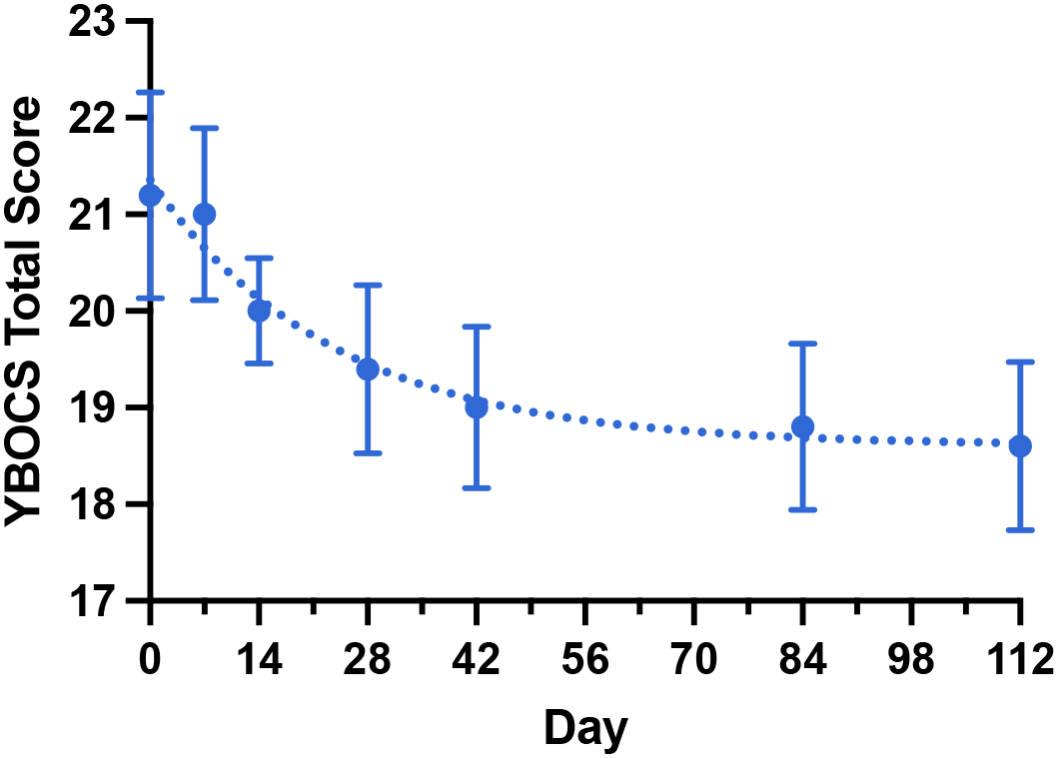
Mean Y-BOCS scores over 112 days of agmatine augmentation (n=5 patients). Data points represent means with standard error of the mean (SEM). Solid line shows exponential decay model fit: Y = (Y0 - Plateau) × exp(-K × X) + Plateau, Y = (Y_0_ - b) × exp(-µ × X) + b, with Y_0_ = 21.36, b = 18.61, µ = 0.04222, t_0.5_ = 16.42 days. Model demonstrates gradual symptom reduction with normality of residuals (D’Agostino-Pearson K2 = 1.209, P = 0.55). The sample size n=5 for all data points.

Mean baseline Y-BOCS score was 21.4 (SEM 0.5, range 17-25). Exponential decay modeling revealed gradual symptom reduction over 112 days (**Figure 1**). Model parameters: Y_0_ = 21.36, b = 18.61, µ = 0.04222, yielding a descriptive half-life t_0.5_ = 16.42 days. Y-BOCS scores demonstrated normality (D’Agostino-Pearson K2 = 1.209, P = 0.55).

**Figure 2:**
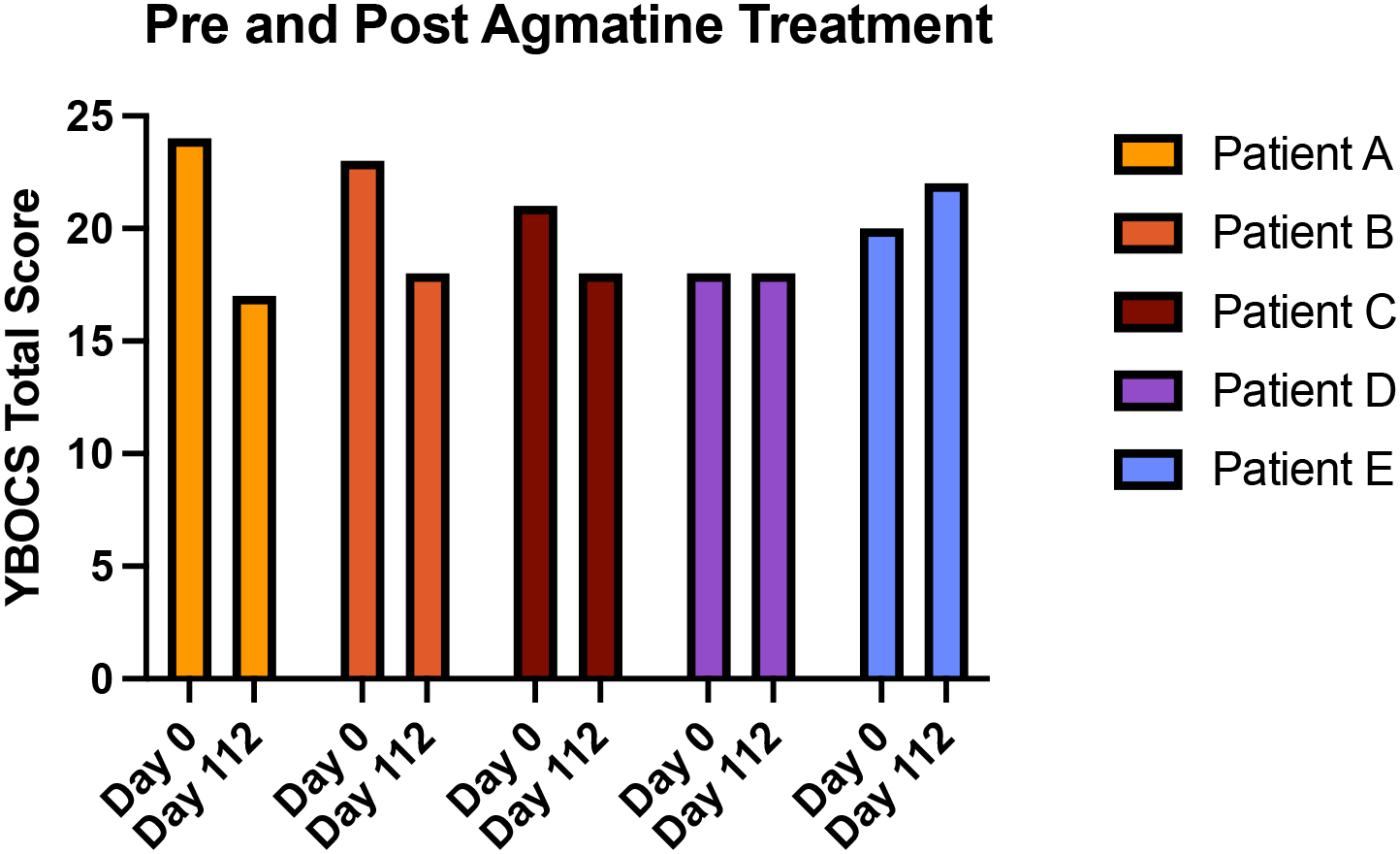
Individual patient Y-BOCS trajectories over treatment course. Patients A, B demonstrated clinically meaningful improvement (≥25% reduction). Patient C showed minimal improvement (~15% reduction). Patients D, E showed no response to agmatine augmentation. Bars represent Y-BOCS scores at baseline compared to the end of the 16-week treatment period.

Individual response patterns varied considerably (**Figure 2**). Two patients (40%; Patients A and B) demonstrated clinically meaningful improvement (≥25% Y-BOCS reduction. Patient A showed the most robust response, with Y-BOCS decreasing from 24 to 17 (30% reduction) by week 16. One patient (20%; Patient C) showed minimal improvement (~15% reduction), suggesting possible non-response. Two patients (40%; Patients D and E) showed no improvement by the end of the study.

### Safety and tolerability

Agmatine was generally well-tolerated with no treatment discontinuations. Two patients (Patients B and D) reported mild transient gastrointestinal symptoms (loose stools, mild nausea) during the first 14 days, which resolved spontaneously without dose adjustment. No serious adverse events, significant laboratory abnormalities, or drug-drug interactions were observed. No patients reported sedation, cognitive impairment, or other central nervous system effects.

## Discussion

This case series provides preliminary evidence supporting the safety and potential efficacy of agmatine augmentation in treatment-resistant OCD. The 40% responder rate and mean Y-BOCS reduction of 2.7 points, while modest, are comparable to established augmentation strategies and consistent with the 25-50% response rates observed with other glutamatergic modulators in the Coelho meta-analysis (8). The gradual response pattern, characterized by a half-life of 16.4 days, contrasts with the rapid effects observed with ketamine but resembles the slower onset typical of SSRI augmentation strategies. Note that the half-life calculated here is descriptive in nature given the small sample size and risks of overfitting.

The observed clinical heterogeneity mirrors broader challenges in glutamatergic augmentation research. Three patients demonstrated clinically meaningful improvements while two showed minimal benefit, suggesting potential response predictors warrant investigation. Baseline symptom severity, specific symptom dimensions (contamination vs. symmetry), and prior glutamatergic agent exposure may influence treatment response. Patient E’s non-response despite previous memantine failure suggests possible NMDA receptor-mediated resistance mechanisms, though cross-tolerance between different NMDA antagonists remains unexplored.

Agmatine’s favorable tolerability profile represents a significant advantage over conventional augmentation strategies. Unlike second-generation antipsychotics, which carry risks of metabolic syndrome, extrapyramidal symptoms, and akathisia (4), agmatine produced only mild transient gastrointestinal effects in 40% of patients. This tolerability advantage may improve treatment adherence and enable longer trial durations necessary to achieve therapeutic benefits given the gradual response pattern observed.

The mechanistic basis for agmatine’s effects likely involves selective GluN2B-containing NMDA receptor antagonism combined with AMPA receptor activation and imidazoline receptor binding (11–13). This multimodal mechanism may provide advantages over single-target glutamatergic modulators, potentially explaining the response in patients who previously failed memantine (Patient E) or NAC (Patient A). The synergistic effects observed in preclinical studies between agmatine and SSRIs (14) support the combination approach employed in this series.

Several limitations warrant consideration. The open-label design without placebo control limits causal inference, as placebo response rates in OCD trials typically range from 5-20% (3). The small sample size (n=5) and absence of formal statistical hypothesis testing preclude definitive efficacy conclusions. The 16-week follow-up period, while adequate for observing initial response patterns, may be insufficient to capture maximal therapeutic effects given the gradual time course observed. Additionally, Y-BOCS assessments conducted by the treating clinician may introduce bias despite the validated nature of the instrument.

Because agmatine carries numerous mechanisms of action, including NMDA antagonism, imidazoline binding, and nitric oxide modulation, it remains unclear whether the failure of response in some subjects is due to NMDA resistance or another pathway such as nitric oxide modulation effects. This is particularly important given the hypothetical link between nitric oxide modulation and OCD treatment response that has been previously discussed. Moreover, the limited sample size renders it difficult to state with certainty whether the lack of response in some subjects was due to pharmacological failure or an idiosyncratic lack of response. Finally, the open-label design, lack of a control group, and clinician-rated outcome assessments render it impossible to generate causal inference, particularly given the high potential for placebo effects and other nonspecific clinical factors.

Future research should employ randomized, double-blind, placebo-controlled designs with adequate sample sizes to definitively establish agmatine’s efficacy in treatment-resistant OCD. Dose-response studies are needed to optimize therapeutic dosing, as the 1300 mg/day dose employed represents an empirically derived starting point based on safety data from non-psychiatric populations (15). Longer follow-up periods (6–12 months) would clarify durability of response and long-term safety. Investigation of potential biomarkers—including baseline glutamate concentrations via magnetic resonance spectroscopy, genetic polymorphisms in GRIN2B or other glutamatergic genes, and symptom dimension profiles—may identify patients most likely to benefit from agmatine augmentation.

Notably, we employed a rather low dose of agmatine (1300 mg/d) compared to the much greater doses used in human studies of neuropathic pain. It is possible that higher doses of agmatine may further improve outcomes, and we recommend a comprehensive dose-escalation study to assess treatment outcomes in future endeavors.

These larger studies should also employ rigorous monitoring of blood pressure, heart rate, and basic laboratory results during dose escalation periods, given that agmatine’s modulation of nitric oxide synthase can impact hemodynamics. We did not rigorously monitor hemodynamics in this study, which remains a limitation that can be addressed in future, larger trial designs.

This case series demonstrates that agmatine augmentation merits further investigation as a novel, well-tolerated strategy for treatment-resistant OCD. The 40% responder rate, favorable safety profile, and mechanistic rationale support advancement to controlled trials. Given substantial unmet need in treatment-resistant OCD populations and limited augmentation options beyond antipsychotics, agmatine represents a promising therapeutic candidate that could meaningfully expand the treatment armamentarium for this disabling condition.

## Data Availability

The original contributions presented in the study are included in the article/Supplementary Material. Further inquiries can be directed to the corresponding author.
